# Robotic simple prostatectomy plus panniculectomy and giant umbilical hernia repair

**DOI:** 10.1590/S1677-5538.IBJU.2018.0565

**Published:** 2019-07-27

**Authors:** Angelica Beatriz Hernandez, Luis G. Medina, Pierre A. Hueber, Felipe Placco Araujo Glina, Hannah Landsberger, Daniel Oberlin, Giovanni Cacciamani, Byron Lopez, Ketan Patel, Rene J. Sotelo

**Affiliations:** 1 Department of Urology, Keck School of Medicine, University of Southern California, Los Angeles, CA, USA; 2 Department of Surgery, Division of Plastic and Reconstructive Surgery, Keck School of Medicine, University of Southern California, Los Angeles, CA, USA

## Abstract

**Introduction:**

Simple prostatectomy is the gold standard for prostates >80 grams, robotic system has proven to help into speed the recovery of the patient and in morbid obesity the advantages of the robotic system can help to perform a successful surgery.

**Case:**

80 years old male with morbid obesity (BMI 45) and several other comorbidities, with history of an umbilical hernia and obstructive lower urinary tract symptoms in acute urinary retention. PSA was 7 ng/dl, DRE demonstrates a >100gr prostate gland. A robotic simple prostatectomy, urethropexy, umbilical hernia repair and panniculectomy in Fleur-de-Lis was performed.

**Results:**

Operative time (OT) and estimated blood loss (EBL) were 438 min and 160 ml respectively. A JP drain was placed in the pelvis and 2 additional were left in the abdominal cavity with several Penrose drains. No immediate or intraoperative complications were observed. The length of stay (LOS) was 6 days without complications. Pathology report showed prostate of 304gr and benign prostatic tissue.

**Discussion:**

In patients with multiple comorbidities robot-assisted surgery provides advantages of shorter LOS, EBL, less transfusion and lower rate of complications. In patients with morbid obesity where the increased girth makes difficult the open approach, robotic surgery is an ideal way to provide definitive treatment; concomitant, Fleur-de-Lis panniculectomy can correct the abdominal contour in both vertical and horizontal orientation at the same time that provides a better plane for trocar insertion, an accurate location of the needle tip and a proper position of the remote center decreasing the possible complication of port placement.

## ARTICLE INFO

**Available at: http://www.intbrazjurol.com.br/video-section/20180565_Hernandez_et_al**

**Int Braz J Urol. 2019; 45 (video #11): 641-641**

